# *Mycobacterium tuberculosis* Zinc Metalloprotease-1 Elicits Tuberculosis-Specific Humoral Immune Response Independent of Mycobacterial Load in Pulmonary and Extra-Pulmonary Tuberculosis Patients

**DOI:** 10.3389/fmicb.2016.00418

**Published:** 2016-03-31

**Authors:** Mani H. Vemula, Rakesh Ganji, Ramya Sivangala, Kiran Jakkala, Sumanlatha Gaddam, Sitaramaraju Penmetsa, Sharmistha Banerjee

**Affiliations:** ^1^Department of Biochemistry, School of Life Sciences, University of HyderabadHyderabad, India; ^2^Department of Immunology, Bhagwan Mahavir Medical Research CenterHyderabad, India; ^3^Department of Genetics, Osmania UniversityHyderabad, India; ^4^Free Chest Clinic, PPM DOTS, Mahavir Hospital and Research CentreHyderabad, India

**Keywords:** tuberculosis (TB), extra-pulmonary tuberculosis (EPTB), pulmonary tuberculosis (PTB), Rv0198c, humoral immunity

## Abstract

Conventionally, facultative intracellular pathogen, *Mycobacterium tuberculosis*, the tuberculosis (TB) causing bacilli in human is cleared by cell-mediated immunity (CMI) with CD4^+^ T cells playing instrumental role in protective immunity, while antibody-mediated immunity (AMI) is considered non-protective. This longstanding convention has been challenged with recent evidences of increased susceptibility of hosts with compromised AMI and monoclonal antibodies conferring passive protection against TB and other intracellular pathogens. Therefore, novel approaches toward vaccine development include strategies aiming at induction of humoral response along with CMI. This necessitates the identification of mycobacterial proteins with properties of immunomodulation and strong immunogenicity. In this study, we determined the immunogenic potential of *M. tuberculosis* Zinc metalloprotease-1 (Zmp1), a secretory protein essential for intracellular survival and pathogenesis of *M. tuberculosis*. We observed that Zmp1 was secreted by *in vitro* grown *M. tuberculosis* under granuloma-like stress conditions (acidic, oxidative, iron deficiency, and nutrient deprivation) and generated Th2 cytokine microenvironment upon exogenous treatment of peripheral blood mononulear cells PBMCs with recombinant Zmp1 (rZmp1). This was supported by recording specific and robust humoral response in TB patients in a cohort of 295. The anti-Zmp1 titers were significantly higher in TB patients (*n* = 121) as against healthy control (*n* = 62), household contacts (*n* = 89) and non-specific infection controls (*n* = 23). A significant observation of the study is the presence of equally high titers of anti-Zmp1 antibodies in a range of patients with high bacilli load (sputum bacilli load of 300+ per mL) to paucibacillary smear-negative pulmonary tuberculosis (PTB) cases. This clearly indicated the potential of Zmp1 to evoke an effective humoral response independent of mycobacterial load. Such mycobacterial proteins can be explored as antigen candidates for prime-boost vaccination strategies or extrapolated as markers for disease detection and progression.

## Introduction

*Mycobacterium tuberculosis*, the tuberculosis (TB) causing bacilli are facultative intracellular parasite, residing primarily in phagocytic cells like alveolar macrophages and monocytes, but may also colonize other cells, such as alveolar epithelial cells, bones, meninges, peritoneal linings of the intestines, etc. (Golden and Vikram, 2005). Traditionally, like all intracellular pathogens, protective immunity to *M. tuberculosis* has been credited to the cell mediated immunity (CMI) with CD4^+^ T cells playing a crucial role in granuloma formation while antibody mediated immunity (AMI) is considered non-protective (van Crevel et al., 2002). The argument that *M. tuberculosis* is strictly intracellular, however, is debatable as *M. tuberculosis*, during some point of its infectious cycle, has also been observed in extracellular spaces (Grosset, 2003), where in principle they can be vulnerable to antibody action. There are a number of reports where despite being intracellular pathogen, antibodies have been shown to modulate the immune response in favor of the host against pathogens (Casadevall, 2003). Studies with antibodies, monoclonal or otherwise, have now demonstrated passive protection for several microbes, such as *Candida albicans* (Han and Cutler, 1995), *Listeria monocytogenes* (Edelson et al., 1999), *Leishmania mexicana* (Anderson et al., 1983), *M. tuberculosis* (Teitelbaum et al., 1998; Zhao et al., 2011) etc., though experiments with immune serum have provided inconsistent results. Similarly, a considerable expanse of data propose that defense against intracellular and extracellular pathogens are not stringently restricted to either Th1 (promoting CMI) or Th2 (promoting AMI) responses. Citing a few examples, humoral immunity have been shown to be protective against intracellular pathogens like Plasmodium or Mycobacteria, while protective immunity against extracellular parasitic flatworm Schistosoma was due to CMI triggered by Th1 response (Abebe and Bjune, 2009; Greenhouse et al., 2011; Wen et al., 2011; Dups et al., 2014). Recently, Modified Vaccinia Ankara 85A (MVA85A) failed to clear the phase 2b trial, where *M. tuberculosis* major secreted antigen complex 85A (Ag85A) that induces a strong Th1 immune response in BCG-primed host was used (Tameris et al., 2013). Therefore, though CMI may remain the mainstream immune response, the role of AMI in conferring protection against intracellular pathogens, including *M. tuberculosis*, cannot be dismissed.

Serological studies advocate that *M. tuberculosis* infection, beside CMI, also evokes a strong humoral response in patients against a variety of mycobacterial antigens (Steingart et al., 2009). Corroborating these observations are other studies where *M. bovis* BCG vaccination led to generation of mycobacterial antigen specific IgG and IgM (Beyazova et al., 1995; Brown et al., 2003; de Valliere et al., 2005). Some anti-*M. tuberculosis* antibodies enhanced both innate and CMI responses during mycobacterial infection (de Valliere et al., 2005). Antibodies, through a range of mechanisms, including simple opsonization to complicated FcR activation can regulate the fate of intracellular pathogens. Some vaccine trials have included induction of AMI to transduce protection against fungal diseases (Vecchiarelli et al., 2012). A recent study has evaluated the feasibility of using humoral immunity in vaccine development against *M. tuberculosis* by comparing immunoglobulin titers (IgG and IgA) with a variety of clinical and immunological parameters (Niki et al., 2015). While these studies strongly support the inclusion of evoking AMI alongside CMI in TB vaccine development program, there is a need for systematic investigation of mycobacterial antigens for a strong and specific humoral response that can be employed against TB.

Surface-exposed or secreted proteins of *M. tuberculosis* are customarily the targets of immune responses in the infected host. Several *M. tuberculosis* proteins, including culture filtrate proteins have been evaluated for their immunogenic properties, such as CFP10, ESAT-6, Ag85B, ICDs etc. (Banerjee et al., 2004; Sinha et al., 2005; Malen et al., 2008; Floss et al., 2010). *M. tuberculosis* GlcB (malate synthase), MPT51 (FbpC1), and HSPX (alpha crystalline) have also been evaluated for humoral response in clinically asymptomatic Health-care workers with latent infections, suggesting the possibility of these responses to be protective (Reis et al., 2009). In this study, we have evaluated the humoral response to *M. tuberculosis* H37Rv zinc metalloprotease-1, Zmp1 (Rv0198c), a protein present in the culture filtrate (de Souza et al., 2011). Extracellular zinc-containing metalloproteases are ubiquitously present, quite a few of them from pathogenic bacteria function as exotoxins, such as, Clostridial neurotoxins, Anthrax toxins, Botulinum neurotoxin, *Bacillus* sp. thermolysin etc. (Hase and Finkelstein, 1993; Miyoshi and Shinoda, 2000). These zinc-metalloproteases are also known to elicit a strong and specific humoral response, for which the inactivated toxin (toxoid) function as vaccine candidate (Hase and Finkelstein, 1993; Miyoshi and Shinoda, 2000). The annotated zinc-metalloproteases from *M. tuberculosis* H37Rv are, namely, Rv0198c (zmp1), Rv0563 (htpX), Rv2467 (pepN), Rv2869c (rip) and Rv3610c (ftsH), Rv1977 (Stewart et al., 2002; Griffin et al., 2011; Kelkar et al., 2011; Mazandu and Mulder, 2012; Schneider et al., 2014). Mutant studies of Zmp1 in *M. tuberculosis* strain H37Rv and *M. bovis* BCG suggested that it is essential for the intracellular survival of the bacteria and possibly impairs inflammasome activation and phagosome maturation (Master et al., 2008; Johansen et al., 2011). With these cues suggesting Zmp1 as an immunomodulator and upon recording high antigenic index by Jameson-Wolf plot using Protean software, we hypothesized that Zmp1 could be a strong immunostimulant and provoke an effective humoral response during infection. Presence of anti-Zmp1 antibodies in TB patient sera confirmed that it is indeed expressed during infection. We established that Zmp1 is secreted by H37Rv under granuloma-like *in vitro* growth conditions and is capable of orienting the immune response toward Th2 cytokine microenvironment. Further, we compared the humoral response to Zmp1 in various TB patient categories, including smear-negative Extra-pulmonary TB cases. Presence of high titers of anti-Zmp1 antibodies in smear-negative extra-pulmonary tuberculosis (EPTB) cases similar to pulmonary tuberculosis (PTB) patients that ranged from low to high bacillary load indicated the potential of Zmp1 to evoke an effective humoral response independent of mycobacterial load. The study points to the potential of further exploration of mycobacterial proteins, such as Zmp1, as antigen candidates for prime-boost vaccination strategies or as markers for disease progression.

## Materials and Methods

### Cloning, Expression, and Purification of Zmp1 (rZmp1) Protein

**FIGURE 1:**
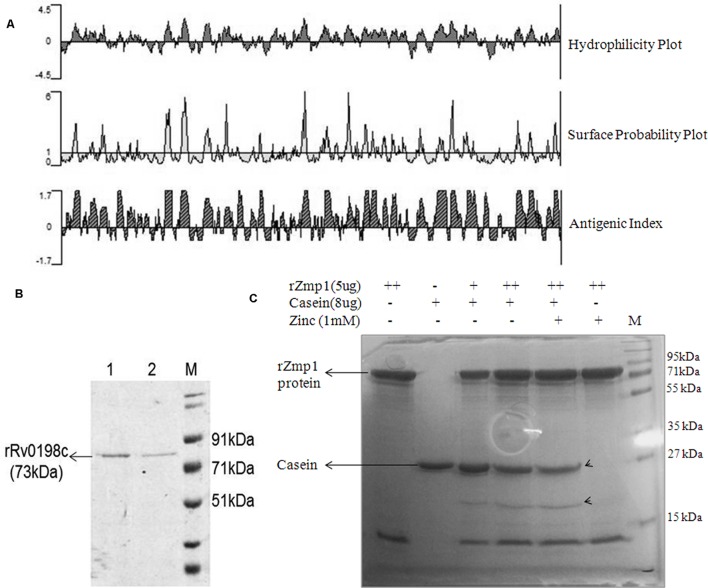
**Rv0198c (Zmp1) protein is antigenic and purified recombinant Zmp1 (rZmp1) was functionally active.** **(A)** Antigenicity prediction of Rv0198c using PROTEAN software. Kyte-Doolittle plot for hydrophilicity; Emini plot for Surface probability and Jameson-Wolf analyses for Antigenic index. **(B)** SDS-PAGE showing purified recombinant Rv0198c protein (rRv0198c), M: marker. Rv0198c was cloned in pET28a (+) with N-terminal 6× Histidine tag and expressed in *Escherichia coli* BL21-DE3 and purified using Cobalt-based affinity chromatography. **(C)** SDS-PAGE depicting the functional activity of purified rRv0198c using casein as substrate. Endotoxin-free rRv0198c was incubated with casein in presence or absence of Zinc ions. Casein was hydrolyzed by rZmp1 yielding two bands (marked by arrowheads) corresponding to apparent molecular weights of 27 and 20 kDa.

The antigenic index of Rv0198c was analyzed by Protean software (Protean 5.00, DNASTAR, Inc.; **Figure 1A**). Rv0198c *(zmp1)* gene was amplified from the genomic DNA of H37Rv using specific forward (5′-ataGGATCCgtgacacttgccatcccctcgg-3′) and reverse (5′-agtCTCGAGgtcttagcctagttccagat-3′) primers. The amplicon was cloned into *Bam*HI and *Xho*I sites of pET28a vector. The positive clones were confirmed by sequencing. pET28a*-Rv0198c* construct was expressed in BL-21 DE3 cells and N-terminal Histidine tagged recombinant Zmp1 (rZmp1) was purified under native conditions (50 mM Tris-HCl buffer, 300 mM NaCl, pH-8.0) by affinity chromatography using cobalt based resin. The purified protein was dialyzed against dialysis buffer (50 mM Tris-HCl buffer pH-8.0, 100 mM NaCl, 4% glycerol, 1 mM PMSF) at 4°C. Dialyzed protein was treated with polymyxin-B agarose beads to remove endotoxins (**Figure 1B**). The endotoxin-free rZmp1 protein was checked for functional activity using casein as a substrate (Rowland et al., 1997; Coffey et al., 2000; Grandgenett et al., 2007). Proteolytic degradation of casein can be assessed on SDS-PAGE by monitoring for cleavage products at lower molecular weights or apparent shift in the casein band to lower molecular weight after proteolytic digestion. Hydrolysis of casein by rZmp1 was performed at 37°C for 1 h in Tris-HCl buffer (50 mM Tris-HCl pH-8.0 and 100 mM NaCl). The reaction was stopped by adding Laemmli’s buffer to the reaction mix followed by fractionation on SDS-PAGE. As anticipated, hydrolysis of casein by rZmp1 has yielded two bands corresponding to apparent molecular weights of 27 and 20 kDa (**Figure 1C**). Band corresponding to 20 kDa is the cleaved product of casein which was observed when casein was incubated with rZmp1 suggesting that the purified protein was functionally active (**Figures 1B,C**).

### *In Vitro* Mycobacterial Growth Conditions and Western Blots

The mycobacterial strain used was *M. tuberculosis* H37Rv. The growth of mycobacteria was performed as described earlier (Ganji et al., 2016). The mycobacteria were plated on 7H10 agar media supplemented with 10% oleic acid, albumin, dextrose, and catalase (OADC) and incubated at 37°C. The colonies were picked into the 7H9 broth media supplemented with 10% OADC and incubated at 37°C at 180 rpm until the OD_600 nm_ reached 0.5–0.6. The culture was checked for any contamination using Ziehl-Neelsen (ZN) staining procedure. The culture was then centrifuged at 3700 rpm for 7 min. The culture pellet was washed with phosphate buffered saline (PBS) pH 7. The pellet was then resuspended in Sauton’s media under granuloma-like stress conditions, such as acidic pH 5.5, oxidative stress (10 mM H_2_O_2_; Voskuil et al., 2011), Iron deprivation and Nutrient starvation for 36 h. For Nutrient stress the culture was resuspended in PBS. For Iron deprivation, the glassware and the media were made Iron-free as described earlier (Hall and Ratledge, 1982).

### Sample Collection

A total of 295 subjects in the age group of 15–60 years were recruited at Mahavir Hospital and Research Centre (MHRC), Hyderabad and University of Hyderabad (UH) after taking prior ethical committee approvals (ECR/450/Inst/AP 2013 and UH/IEC/2014/36) and written consents from the subjects. 2–5 mL of blood was collected in vacutainers with EDTA and later sera were separated for the experiments. The study population was divided into four categories, namely, Clinically Healthy donors (*n* = 62), TB patients (*n* = 121), household contacts (*n* = 89), and non-specific infection controls (*n* = 23). Clinically healthy donors had no symptoms of any disease at the time of sera collection and were tested for TB-interferon-gamma release assays (TB-IGRAs) using QuantiFERON-TB Gold (QFT) ELISA kit (Reference# 0594-0201) and the results were analyzed using QuantiFERON-TB Gold Analysis software (Version 2.62) as per the manufacturer’s instructions (**Supplementary Table S1**). For TB patients, the sputum microscopy for AFB was performed as per Revised National Tuberculosis Control Programme (RNTCP), government of India, guidelines with confirmed diagnosis of sputum, culture, and chest X-ray in patients (http://tbcindia.nic.in/view.php?lid=3143&type=1). Tuberculin skin test (TST) was performed in all the subjects. TB patients were further categorized into PTB (*n* = 66) and EPTB (*n* = 55) cases. EPTB patients were defined atleast with one culture-positive specimen from an extra-pulmonary site, or histological or radiological, or strong clinical evidence consistent with active extra-pulmonary TB. Household contacts of the respective patients were those who resided in house of the TB patient during 3 months period for atleast seven consecutive days prior to the diagnosis of tuberculosis. Mostly they were siblings and spouses. The household contacts, though, were clinically asymptomatic but many of them were Mantoux positive (Out of 89 household contacts, 60 subjects were Mantoux positive with diameter of Induration >15 mm). Non-specific infection controls comprised of patients with random infections other than TB such as viral, bacterial, and parasitic infections. Pregnant women, terminally ill patients, immunocompromised patients, patients undergoing any chemotherapy or with chronic illness were not included in the study.

### Enzyme Linked Immunosorbent Assay

The peripheral blood mononulear cells (PBMCs) were isolated from blood collected from healthy donors using Ficoll gradient. They were either left untreated or exogenously treated with 50 and 100 nM of purified, endotoxin-free rZmp1 or 0.5 μg/mL of LPS in RPMI media supplemented with 10% FBS and kept at 37°C for 24 h. For measurement of cytokine levels we have used BD OptEIA enzyme linked immunosorbent assay (ELISA) sets and performed as per manufacturer’s instructions. For measurement of anti-Zmp1 antibody titers, 100 ng of purified rZmp1 in 100 μL of coating buffer was coated per well at 4°C overnight. The plates were then washed three times with PBST (PBS containing 0.05% Tween-20) before blocking with 120 μL of blocking solution (3% BSA in PBS) at 37°C for 1 h. After blocking, plates were washed thrice with PBST. 1:100 times diluted (100 μL/well) sera samples were added and incubated at 37°C for 1 h followed by five washes with PBST. Anti-Human IgG conjugated with HRP (Sigma) was used as the secondary antibody in 1:10000 dilution (100 μL/well) and incubated at 37°C for 1 h. After seven washes with 1X PBST, 100 μL/well of chromogenic substrate (Tetramethylbenzidine) was added and kept for incubation at 37°C for 30 min. The reactions were stopped using 100 μL of 2 N H_2_SO_4_. The absorbance was measured at 450 and 570 nm in multi-well plate reader (Biotek). To check for the cross-reactivity of rZmp1 with other anti-TB antibodies, we have performed Western blotting for the rZmp1 using anti-ESAT6 antibody (**Supplementary Figure S1**). rZmp1 is not detected by the anti-ESAT6 antibody in the Western blot (**Supplementary Figure S1**) and thus confirms the specificity of rZmp1 and fidelity of rZmp1-based ELISA method to determine the anti-Zmp1 antibody titers in the sera samples.

### Graphs and Statistical Analyses

Statistical analyses were carried out using SigmaPlot software version 11.0.0.77 (Systat Software, Inc., USA). For cytokine data, One-way ANOVA was performed with Holm–Sidak multiple pair-wise comparison method and the threshold for significance was set at *p* < 0.05. The error bars represent the ± standard deviation (SD) from the mean of at least three independent experiments. For statistical analyses of anti-Zmp1 antibody titers measured from the blood samples, One-way ANOVA on ranks was performed with Dunn’s method for pair-wise comparison method. The threshold for significance was set at *p* < 0.05. They were represented as box plots using SigmaPlot. Within the plots, the upper quartile of the box represents the 75th percentile and the lower quartile for the 25th percentile. The line inside the box represents the median. The whiskers arising from either side of the upper half and the lower half of the box correspond to 1.5 times the interquartile range (IQR; Benjamin et al., 2013). Any datum to the further extreme of the whiskers is termed as outlier.

## Results

### Zmp1 Protein is Expressed during Infection and is Secreted under Granuloma-Like *In Vitro* Growth Conditions by *M. tuberculosis* H37Rv

**FIGURE 2:**
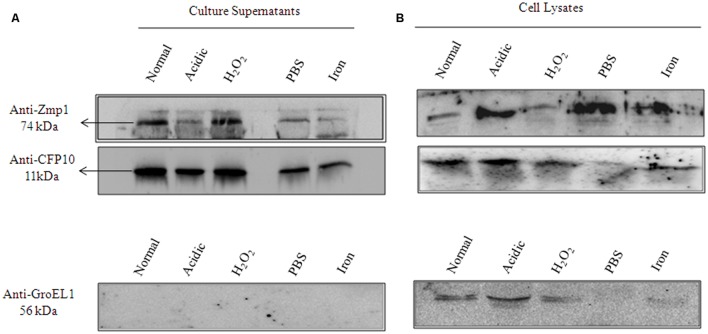
**Zmp1 is expressed and secreted by *in vitro* grown *Mycobacterium tuberculosis* H37Rv under granuloma-like conditions.** **(A)** The culture supernatants of *in vitro* grown *M. tuberculosis* H37Rv under normal conditions and under various stress conditions, such as, acidic pH5.5, H_2_O_2_ induced oxidative stress, nutrient starvation and iron depletion, were precipitated by 10% trichloroacetic acid and the resultant precipitate was subjected to Western blot using Mouse anti-Zmp1 antibody (1:1000 dilution; Upper panel). Rabbit anti-CFP10 antibody (1:1000 dilution) against CFP10, a culture filtrate protein 10, was used as a positive control (Middle Panel) and Mouse anti-GroEL1 antibody (1:1000 dilution) against GroEL1, a cytoplasmic chaperone was used as a negative control (Lower Panel) to evaluate for cell lysis products in supernatants. The absence of band corresponding to GroEL1 in the supernatant fractions suggests the purity of culture filtrate preparations. **(B)** Figure represents the Western blots with the whole bacterial lysates as control experiment.

The functionally active, endotoxin free, purified recombinant Zmp1 (rZmp1) was used as antigen to capture anti-Zmp1 antibodies in the sera samples of TB patients (results presented and discussed later). The presence of anti-Zmp1 antibodies in *M. tuberculosis* infected patients confirmed that Zmp1 was indeed expressed by *M. tuberculosis* during infection. Zmp1 was identified as one of the culture filtrate proteins of *in vitro* grown H37Rv (de Souza et al., 2011). We extended the study to check if the same holds true for granuloma-like conditions. To study the same, H37Rv was grown under different stress conditions known to simulate acellular caseous environment of TB granulomas, that is, acidic pH 5.5, H_2_O_2_ induced oxidative stress, nutrient deprivation and iron deficiency (Stallings and Glickman, 2010). Bacteria were grown to mid-log phase and then subjected to various stresses for 36 h. The mycobacterial cells were then harvested and the culture supernatants separated. Culture supernatants were then precipitated using 10% TCA and the precipitate was used to detect presence of Zmp1 protein using Western blotting with in-house generated anti-Zmp1 antibody (**Figure 2A**, upper panel). CFP10 and GroEL1, which were probed with their respective antibodies, were used as positive and negative controls respectively (**Figure 2A**, middle and lower panel). CFP10 is a known mycobacterial secretory protein (Malen et al., 2007, 2008) and hence was used as a positive control for culture supernatant preparations while GroEL1 is an intrabacterial, membrane associated protein which is not secreted out (de Souza et al., 2011; Malen et al., 2011) and is used as negative control. Absence of GroEL1 in the culture supernatants indicated absence of cell lysis products in the culture supernatant (**Figure 2A**, lower panel). The presence of band corresponding to Zmp1 in Western blots suggested secretion of the protein under all the tested stress conditions (**Figure 2A**, upper panel). The assay was a qualitative check to confirm the secretion of Zmp1, though it is possible that the levels of secreted Zmp1 in culture supernatants may vary with stress conditions. **Figure 2B** represents the Western blots with the whole bacterial lysates as control experiment. This suggested that Zmp1 is indeed a secreted protein, possibly secreted within granuloma of infected host.

### rZmp1 Stimulated PBMCs to Release Th2 Class of Cytokines

We next evaluated the immunostimulatory potential of Zmp1 in terms of release of Th1/Th2 cytokines from exogenously treated PBMCs derived from healthy volunteers to elucidate the association of Zmp1 with CMI or AMI. To do the same, functionally active, endotoxin free rZmp1 was used for stimulatory assays.

**FIGURE 3:**
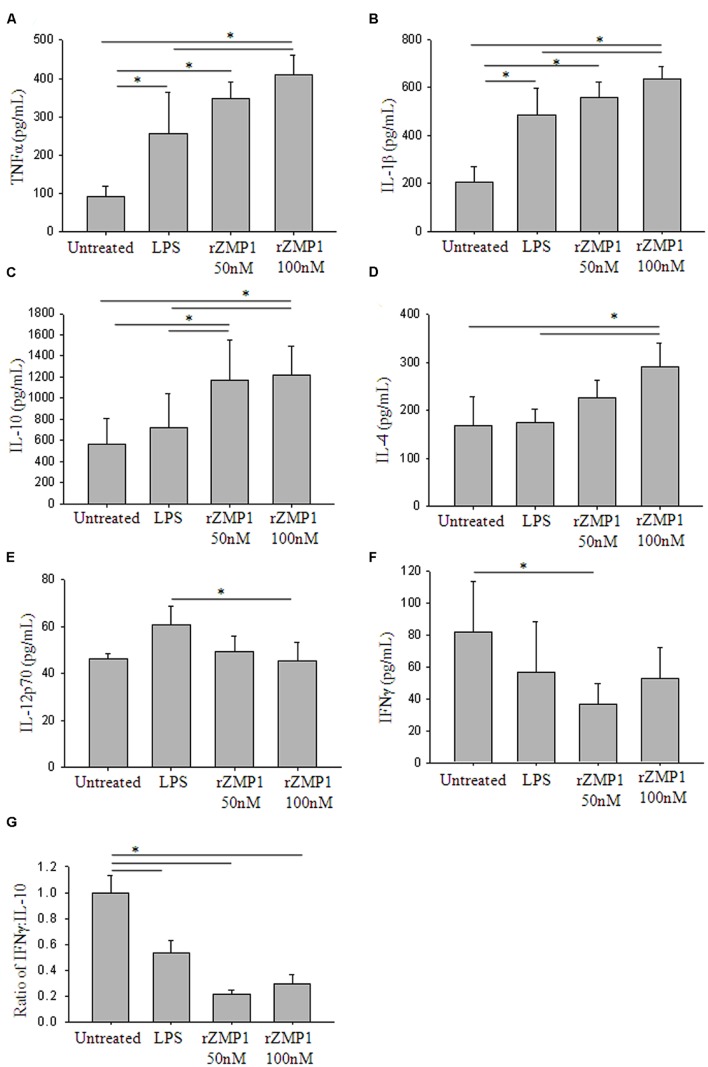
**Treatment of PBMCs with recombinant Zmp1 protein inclines the immune status to Th2 response.** Cytokine profiles of the PBMCs either untreated or treated with LPS, 50 nM rZmp1 and 100 nM rZmp1 was estimated. **(A)** TNFα, **(B)** IL-1β, **(C)** IL-10, **(D)** IL-4, **(E)** IL-12p70, **(F)** IFNγ titers were measured using capture ELISA. **(G)** Ratio of IFNγ:IL-10. All the experiments were performed more than three times. Statistical analyses were done using one-way ANOVA with Holm-Sidak multiple pair-wise comparison method. Error bars represent ±SD (standard deviation). ^∗^ Represents *p* < 0.05.

Peripheral blood mononulear cells were treated with rZmp1 at 50 and 100 nM for 24 h. LPS, a known strong immunostimulant of PBMCs, was used as a positive control (Jansky et al., 2003). The culture supernatants were then collected to assay for the levels of a minimal battery of cytokines. TNF-α and IL-1β are the cytokines of innate response that stimulate the acute phase reaction and represent initial stimulation of immune cells. High titers of TNF-α (Untreated: 91.98 ± 27.67 pg/mL; rZmp1 100 nM: 408.71 ± 52.96 pg/mL) and IL-1β (Untreated: 205.62 ± 65.63 pg/mL; rZmp1 100 nM: 634.34 ± 51.72 pg/mL; **Figures 3A,B**) upon exogenous treatment of PBMCs with rZmp1 established that rZmp1 is indeed a strong immunostimulant, comparable with LPS (TNF-α: 255.89 ± 107.67 pg/mL; IL-1β: 485.46 ± 111.66 pg/mL; **Figures 3A,B**). We next measured the levels of pro-inflammatory cytokines IFN-γ and IL-12p70 and regulatory cytokines IL-4 and IL-10. It was observed that upon treatment of PBMCs with rZmp1 protein, there is an increased secretion of regulatory cytokines, IL-10 (Untreated: 562.41 ± 244.02 pg/mL; rZmp1 100 nM: 1218.77 ± 270.60 pg/mL) and IL-4 (Untreated: 168.91 ± 60.62 pg/mL; rZmp1 100 nM: 292.31 ± 49.13 pg/mL; **Figures 3C,D**) and while no change was observed in the levels of IL-12p70 (Untreated: 46.32 ± 2.15 pg/mL; rZmp1 100 nM: 45.23 ± 8.31 pg/mL) and IFNγ (Untreated: 81.84 ± 31.44 pg/mL; rZmp1 100 nM: 52.49 ± 19.43 pg/mL; **Figures 3E,F**). When the ratio of IFNγ to IL-10 was considered, rZmp1 treatment showed lower IFNγ:IL-10 ratio of 0.30 ± 0.07 as compared to untreated (1 ± 0.13) or LPS (0.54 ± 0.10) treated (**Figure 3G**). This indicated that rZmp1 promoted a Th2 immune response. Summing up, these experiments indicated that the secreted *M. tuberculosis* Zmp1 should incline the immune system toward Th2 response, promoting humoral immunity.

With the above experiments indicating that Zmp1 induces Th2 response, we next assessed if this is manifested in the form of anti-Zmp1 antibody production in TB patients. To verify the same, we measured anti-Zmp1 antibodies in the sera samples of TB patients using rZmp1 as the bait antigen in ELISA based assays.

### Zmp1 Elicited a Strong B-cell Response Which Was Specific for Tuberculosis (TB) Infection

**FIGURE 4:**
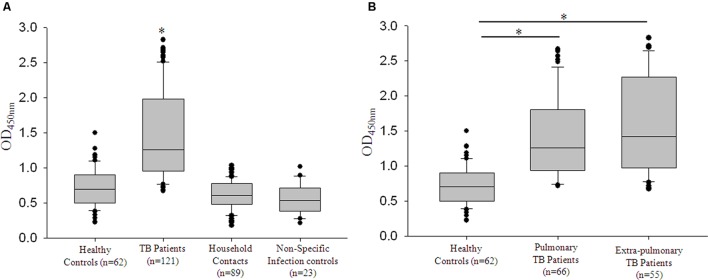
**rRv0198c ELISA was specific for tuberculosis (TB) infection.** **(A)** Box plots representing titers of anti-Rv0198c antibody titers as indicated by values at Absorbance 450 nm in Healthy, TB, Household or patient contacts and Non-specific infection control. ^∗^The differences in Abs450 values for TB vs. Healthy; TB vs. Household contacts and TB vs. non-specific infection controls were highly significant and a *p*-value of <0.001 was observed in all the three cases. **(B)** The box plots representing the anti-Rv0198c antibody titers in Healthy controls, Pulmonary TB patients and Extra-pulmonary TB patients. Statistical analyses were done using one-way ANOVA on ranks was performed with Dunn’s method for pair-wise comparison method. ^∗^ Represents *p* < 0.05.

The humoral response of the host against mycobacterial secretory protein, Zmp1 was scored in a study population comprising 295 subjects. This included four groups, TB patients, Healthy controls, Household contacts of TB patients and volunteers with non-specific infections. Purified rZmp1 was used as bait antigen in the indirect ELISA to score for the anti-Zmp1 antibodies in the serum samples of healthy (*n* = 62), TB patients (*n* = 121), household contacts (*n* = 89), and non-specific infection control samples (*n* = 23; **Figure 4A**). We observed that there was a significant increase (*p* < 0.001) in the absorbance at 450 nm corresponding to the anti-Zmp1 antibody titers in TB patient sera as compared to the healthy or household contacts or non-specific infection controls (**Figure 4A**). The median values for the Healthy was 0.702 (IQR: 0.506–0.893), TB patients was 1.264 (IQR: 0.961–1.982), household contacts was 0.606 (IQR: 0.486–0.78) and non-specific infection control was 0.539 (IQR: 0.394–0.708) showing distinct difference of TB patients from other groups. The non-reactivity of rZmp1 to sera samples of non-specific infection controls clearly indicated that the rZmp1 did not cross-react with the antibodies generated due to other infections in human host. In addition, negligible absorbance observed in sera of healthy donors and Household contacts strongly points to the specificity of rZmp1 to TB infection. This clearly indicated that Zmp1 could elicit a strong humoral response that was specific to TB patients and could clearly distinguish TB patient category from all other categories, including asymptomatic household contacts.

### Zmp1 Elicits Strong B-cell Response Independent of Mycobacterial Load in TB Patients

Having seen that rZmp1 stimulated release of Th2 cytokines over Th1 in PBMCs and accordingly Zmp1 elicited a strong B-cell response that could be detected in terms of high anti-Zmp1 IgG titers in TB patients, we next checked if these titers differed between PTB (*n* = 66) and EPTB (*n* = 55) cases. This was important as all EPTB patients were sputum smear-negative but recorded presence of mycobacteria by acid-fast bacilli (AFB) staining and caseous necrosis in their biopsy samples, suggesting localized mycobacterial load. We observed that the median values for both PTB and EPTB cases were approximately similar, viz.; for PTB it was 1.257 (IQR: 0.956–1.8) and for EPTB, it was 1.421 (IQR: 0.979–2.259; **Figure 4B**). We then compared the anti-Zmp1 titers in PTB patients with various gradients of mycobacterial load. **Table 1** lists the representative ELISA readings of PTB patients ranging from a high bacilli load (3+) to smear negative, synonymous with extremely low bacilli titers, clearly showing humoral response to Zmp1 was independent of *M. tuberculosis* load. This observation was significant, as it suggested that Zmp1 could elicit a strong humoral response even in paucibacillary PTB cases and can detect EPTB cases significantly.

**Table 1:** Representative table to show that pulmonary TB (PTB) cases with varying loads of bacilli load in sputum does not show variation in their Abs_450 nm_ when detected by rZmp1 ELISA test.

Sample no.	AFB (Bacilli load)	Abs_450_
PTB# 1	PI -VE	1.260667
PTB# 2	PI -VE	1.297333
PTB# 3	PI -VE	1.407333
PTB# 4	PI -VE	1.716
PTB# 5	PI -VE	1.936333
PTB# 6	PI -VE	2.3335
PTB# 7	PI 1+	1.127333
PTB# 8	PI 1+	1.178333
PTB# 9	PI 1+	1.725667
PTB# 10	PI 1+	1.978333
PTB# 11	PI 1+	2.317333
PTB# 12	PI 2+	1.249667
PTB# 13	PI 2+	1.319667
PTB# 14	PI 2+	1.773
PTB# 15	PI 2+	1.800333
PTB# 16	PI 2+	2.487
PTB# 17	PI 3+	1.107
PTB# 18	PI 3+	1.274333
PTB# 19	PI 3+	1.564667
PTB# 20	PI 3+	2.67


*PI 1+ indicates more than 100 acid-fast bacilli (AFB) in sputum, PI 2+ indicates more than 200 acid-fast bacilli in sputum, PI 3+ indicates more than 300 acid-fast bacilli in sputum from 100 microscopic fields, PI -VE indicates no acid-fast bacilli in sputum.*

### Humoral Response to Zmp1 Was Detected Only in Active TB Cases and Not in Their Household Contacts

**FIGURE 5:**
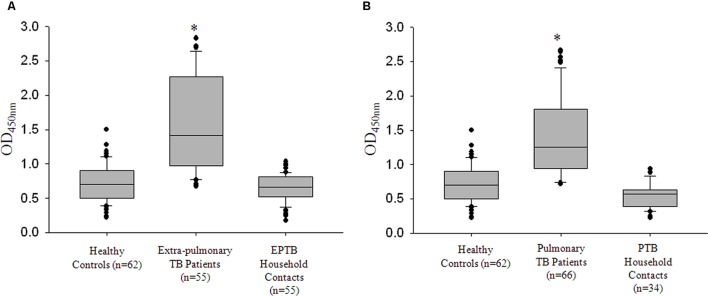
**rRv0198c ELISA detected all active TB cases with high specificity and sensitivity.** **(A)** Box plots representing titers of anti-Rv0198c antibody titers as indicated by values at Absorbance 450 nm in Healthy, extra-pulmonary TB (EPTB), EPTB patient contacts (Household contacts of EPTB patients). **(B)** Box plots representing titers of anti-Rv0198c antibody titers as indicated by values at Absorbance 450 nm in Healthy, pulmonary TB (PTB), PTB patient contacts (Household contacts of PTB patients). ^∗^The differences in Abs450 values for PTB vs. Healthy; PTB vs. PTB Household contacts; EPTB vs. Healthy; EPTB vs. EPTB Household contacts were highly significant and a *p*-value of <0.001 was observed in all the cases. Statistical analyses were done using one-way ANOVA on ranks was performed with Dunn’s method for pair-wise comparison method.

Some of the reports have indicated antibodies against specific *M. tuberculosis* antigens in the sera of clinically healthy, latently infected Health-care workers (Reis et al., 2009). With the anti-Zmp1 antibodies detected in active EPTB and even in active paucibacillary PTB cases (**Table 1**), we wanted to check if anti-Zmp1 titers were also detectable in the respective household contacts. These household contacts had stayed with active patients for at the least seven consecutive days during the 3 months prior to the diagnosis of TB. They were expected to be exposed to *M. tuberculosis* though it is reported that EPTB patients, specifically those with tissue TB, are unlikely to transmit the bacilli. Most of these clinically asymptomatic contacts were tested positive for Mantoux’s test (Out of 89 household contacts, 60 subjects were Mantoux positive with diameter of Induration >15 mm) and hence may represent possible cases of latent TB. To evaluate that, ELISA readings of EPTB (*n* = 55) and their respective household contacts (*n* = 55) and PTB (*n* = 66) and their household contacts (*n* = 34) were plotted (**Figures 5A,B**). It was observed that compared to EPTB patient contacts (Median: 0.663; IQR: 0.524–0.808) or PTB patient contacts (Median: 0.570; IQR: 0.387–0.630), titers of anti-Zmp1 antibody were distinctly high in EPTB patients (Median: 1.421; IQR: 0.979–2.259; *p* < 0.001) or PTB patients (Median: 1.257; IQR: 0.956–1.8; *p* < 0.001), respectively (**Figures 5A,B**), suggesting that Zmp1 humoral response is restricted to active infection cases as against asymptomatic household contacts including Mantoux positive cases under the category which may be possible latent subjects.

## Discussion

Till recently, TB vaccine program was driven by the belief that protective immunity against *M. tuberculosis* infection is chiefly because of CMI generated by Th1 microenvironment. The majority of candidate vaccines were focused on improving CMI either by engineering the present BCG vaccine or using mycobacterial antigens that elicited Th1 responses as boosters after BCG priming. Interestingly, the vaccine strategies that use the whole cell (such as *M. indicus pranii*) has reached phase III of clinical trials (Weiner and Kaufmann, 2014; Tye et al., 2015). The success of this may be owing to a balanced stimulation of both CMI and AMI wings rather than trying to bias the same toward CMI. Additionally, with monoclonal antibodies conferring passive immunity against several intracellular pathogens, including *M. tuberculosis*, humoral response to TB is being explored with a new enthusiasm.

In this study, we deliberated on the immunomodulatory function of a secreted *M. tuberculosis* protein Zinc metalloprotease-1. *M. tuberculosis* Zmp1 is a well characterized protein that alters phagosome maturation and is considered essential for intracellular survival of *M. tuberculosis*. With the X-ray structure available, this protein has been screened for small molecule inhibitors to evaluate it as a potential drug target (Ferraris et al., 2011; Mori et al., 2014). Zmp1 works optimally under slightly acidic conditions with neuropeptides as possible substrates (Petrera et al., 2012). It was also shown to be involved in inflammasome activation (Master et al., 2008), suggesting that it definitely has immunomodulatory function besides its role in regulating phagosome maturation. Protein sequence analyses by Jameson-Wolf plot using Protean software, which recorded high antigenicity indices based on surface probability and hydrophobicity of its amino-acid sequence of Zmp1 further supported the notion that apart from an active enzyme, it can also be a B-cell stimulant (**Figure 1A**).

We confirmed the secretory nature of Zmp1 in granuloma-like *in vitro* growth conditions and concluded that Zmp1 could indeed be released out in the extracellular milieu when *M. tuberculosis* is growing in acidic, nutrient deprived and oxidatively stressed acellular environment at the center of TB granulomas (**Figure 2**). In addition to that, we also observed that it is a strong immune-stimulant and could stimulate mono-nuclear cells to release high titers of TNF-α and IL-1β, the shock inducing cytokines that also start the innate mechanisms toward acute phase reaction. However, subsequently, it was not the pro-inflammatory, but Th2 cytokines that were pre-dominantly released upon rZmp1 stimulation of PBMCs (**Figure 3**). Corroborating the Th2 response, in a cohort of about 121 TB patients, high titers of anti-Zmp1 antibodies could be recorded (**Figure 4**). This study revealed yet another facet of *M. tuberculosis* Zmp1 as a highly immunogenic mycobacterial antigen that could elicit strong and specific humoral response in TB patients.

There are many reports analyzing the levels of IgG antibody titers against *M. tuberculosis* antigens, secretory or otherwise, in different clinical stages (Wu et al., 2010; Baumann et al., 2014, 2015). The studies also claim that these antibodies are present during active disease, but reduce upon decrease in bacterial load with treatment (Singh et al., 2005). That would mean antibody titers are bacterial load dependent and a low bacterial infection may not sufficiently trigger a good humoral response. To check the same, we compared the anti-Zmp1 antibody titers in patients with high or low bacillary loads. To our surprise, anti-Zmp1 titers were equally high in paucibacillary cases as in patient with sputum bacilli score of 3+ (**Figure 5B**; **Table 1**). This suggested that a very low dose of *M. tuberculosis* Zmp1 could induce very strong and specific B-cell response, a property that can be suitably explored for prime–boost vaccination strategy. In total, with the evidence that Zmp1 is secreted by active *M. tuberculosis* in granuloma-like growth conditions and stimulated PBMCs to release Th2 class of cytokines, one may hypothesize that very low concentration of this protein when released in the granulomas of infected host, stimulate the neighboring mono-nuclear cells to eventually generate a Th2 immune response that supports B-cell specific immunity toward Zmp1 resulting in high titers of anti-Zmp1 antibodies in patients. However, unlike reported humoral response to GlcB (malate synthase), MPT51 (FbpC1) and HSPX (alpha crystalline) in latent TB cases (Reis et al., 2009), anti-Zmp1 antibodies could not be detected significantly in healthy household contacts who were asymptomatic, Mantoux’s positive and possibly represent latently infected population in this study.

Likewise, we observed that rZmp1 ELISA was highly specific for TB cases, both PTB and EPTB (**Figure 4B**). The group comprising of non-specific infections were used to rule out if, Zmp1 cross-reacts with antibodies generated against other bacterial or viral proteins. This group had patients with random infections other than TB such as viral, bacterial, and parasitic infections. Non-reactivity of rZmp1 with antibodies in the sera of non-specific infection controls as indicated by low-absorbance values (**Figure 4A**) points to the specificity of rZmp1 to distinctly differentiate TB cases from all other categories. This suggests the potential of rZmp1 ELISA as a disease marker which can be further explored. Identification EPTB disease remains challenging for reasons like diffused symptoms, low *M. tuberculosis* load at the site of infection and difficulties in obtaining clinical specimens from deep-seated organs (Lawn and Zumla, 2012). Dependence on cultures for EPTB frequently leads to substantial delays, compromising patient care and spread of infection to others. Though serological tests are less acceptable in the field of TB detection, the problem possibly lies in the selection of an antigen that shows a good serological response even when infection load is less. In comparison with microscopy and cultures, ELISA based serological tests offer several advantages such as in terms of time, infrastructure and ease of sample collection in the form of peripheral blood.

In this study, we revealed *hitherto* unknown immunogenic property of *M. tuberculosis* Zmp1. Zmp1 is a strong *M. tuberculosis* specific immune-stimulant, the properties of which can be further explored both as a potential vaccine candidate or a disease marker. The possibility of taking the study on antigenicity of Zmp1 to the next level by using larger cohort, blinded samples and multi-centric study is very stimulating from both scientific point of view and translational research.

## Author Contributions

Conceived and designed the experiments: MV, GR, SB. Performed the experiments: MV, RS, KJ, GR, SP. Analyzed the data: MV, GR, RS, KJ, SG, SP, SB. Contributed reagents/materials/analysis tools: SG, SP, SB. Contributed to the writing of the manuscript: MV, GR, SG, SB. We declare that all the authors have approved the article for submission, its contents, order of authorship and that there are no competing interests.

## Conflict of Interest Statement

The authors declare that the research was conducted in the absence of any commercial or financial relationships that could be construed as a potential conflict of interest.

## Supplementary Material

**FIGURE S1 | Cross-reactivity check of anti-Zmp1 antibody and rZmp1.** **(A)** Recombinant mycobacterial antigens, ESAT6 (5 μg), CFP10 (5 μg), and Zmp1 (5 μg) were loaded on to 15% SDS-PAGE followed by Western blot with Mouse anti-Zmp1 antibody. The corresponding Ponceau S stained blot showing transferred protein is provided. **(B)** Recombinant mycobacterial antigens, ESAT6 (5 μg) and Zmp1 (5 μg) were loaded on to 15% SDS-PAGE followed by Western blot with rabbit anti-ESAT6 antibody (1:1000 dilution). The corresponding Ponceau S stained blot showing transferred protein is provided.

## References

[B1] Abebe F. Bjune G. (2009). The protective role of antibody responses during *Mycobacterium tuberculosis* infection. *Clin. Exp. Immunol.* 157 235–243. 10.1111/j.1365-2249.2009.03967.x 19604263 PMC2730849

[B2] Anderson S. David J. R. McMahon-Pratt D. (1983). In vivo protection against *Leishmania mexicana* mediated by monoclonal antibodies. *J. Immunol.* 131 1616–1618. 6619540

[B3] Banerjee S. Nandyala A. Podili R. Katoch V. M. Murthy K. J. Hasnain S. E. (2004). *Mycobacterium tuberculosis* (Mtb) isocitrate dehydrogenases show strong B cell response and distinguish vaccinated controls from TB patients. *Proc. Natl. Acad. Sci. U.S.A.* 101 12652–12657. 10.1073/pnas.0404347101 15314217 PMC514659

[B4] Baumann R. Kaempfer S. Chegou N. N. Oehlmann W. Loxton A. G. Kaufmann S. H. (2014). Serologic diagnosis of tuberculosis by combining Ig classes against selected mycobacterial targets. *J. Infect.* 69 581–589. 10.1016/j.jinf.2014.05.014 24968240

[B5] Baumann R. Kaempfer S. Chegou N. N. Oehlmann W. Spallek R. Loxton A. G. (2015). A subgroup of latently *Mycobacterium tuberculosis* infected individuals is characterized by consistently elevated IgA responses to several mycobacterial antigens. *Mediators Inflamm.* 2015 364758 10.1155/2015/364758 PMC4546975 26347586

[B6] Benjamin R. Banerjee A. Sunder S. R. Gaddam S. Valluri V. L. Banerjee S. (2013). Discordance in CD4^+^T-cell levels and viral loads with co-occurrence of elevated peripheral TNF-alpha and IL-4 in newly diagnosed HIV-TB co-infected cases. *PLoS ONE* 8:e70250 10.1371/journal.pone.0070250 PMC3731333 23936398

[B7] Beyazova U. Rota S. Cevheroglu C. Karsligil T. (1995). Humoral immune response in infants after BCG vaccination. *Tuber. Lung Dis.* 76 248–253. 10.1016/S0962-8479(05)80013-9 7548909

[B8] Brown R. M. Cruz O. Brennan M. Gennaro M. L. Schlesinger L. Skeiky Y. A. (2003). Lipoarabinomannan-reactive human secretory immunoglobulin A responses induced by mucosal bacille Calmette-Guerin vaccination. *J. Infect. Dis.* 187 513–517. 10.1086/368096 12552438

[B9] Casadevall A. (2003). Antibody-mediated immunity against intracellular pathogens: two-dimensional thinking comes full circle. *Infect. Immun.* 71 4225–4228. 10.1128/IAI.71.8.4225-4228.2003 12874297 PMC166024

[B10] Coffey A. van den Burg B. Veltman R. Abee T. (2000). Characteristics of the biologically active 35-kDa metalloprotease virulence factor from *Listeria monocytogenes*. *J. Appl. Microbiol.* 88 132–141. 10.1046/j.1365-2672.2000.00941.x 10735252

[B11] de Souza G. A. Leversen N. A. Malen H. Wiker H. G. (2011). Bacterial proteins with cleaved or uncleaved signal peptides of the general secretory pathway. *J. Proteomics* 75 502–510. 10.1016/j.jprot.2011.08.016 21920479

[B12] de Valliere S. Abate G. Blazevic A. Heuertz R. M. Hoft D. F. (2005). Enhancement of innate and cell-mediated immunity by antimycobacterial antibodies. *Infect. Immun.* 73 6711–6720. 10.1128/IAI.73.10.6711-6720.2005 16177348 PMC1230956

[B13] Dups J. N. Pepper M. Cockburn I. A. (2014). Antibody and B cell responses to *Plasmodium* sporozoites. *Front. Microbiol.* 5:625 10.3389/fmicb.2014.00625 PMC4235289 25477870

[B14] Edelson B. T. Cossart P. Unanue E. R. (1999). Cutting edge: paradigm revisited: antibody provides resistance to *Listeria* infection. *J. Immunol.* 163 4087–4090. 10510340

[B15] Ferraris D. M. Sbardella D. Petrera A. Marini S. Amstutz B. Coletta M. (2011). Crystal structure of *Mycobacterium tuberculosis* zinc-dependent metalloprotease-1 (Zmp1), a metalloprotease involved in pathogenicity. *J. Biol. Chem.* 286 32475–32482. 10.1074/jbc.M111.271809 21813647 PMC3173161

[B16] Floss D. M. Mockey M. Zanello G. Brosson D. Diogon M. Frutos R. (2010). Expression and immunogenicity of the mycobacterial Ag85B/ESAT-6 antigens produced in transgenic plants by elastin-like peptide fusion strategy. *J. Biomed. Biotechnol.* 2010 274346 10.1155/2010/274346 PMC2855997 20414351

[B17] Ganji R. Dhali S. Rizvi A. Sankati S. Vemula M. H. Mahajan G. (2016). Proteomics approach to understand reduced clearance of mycobacteria and high viral titers during HIV-mycobacteria co-infection. *Cell. Microbiol.* 18 355–368. 10.1111/cmi.12516 26332641

[B18] Golden M. P. Vikram H. R. (2005). Extrapulmonary tuberculosis: an overview. *Am. Fam. Physician* 72 1761–1768. 16300038

[B19] Grandgenett P. M. Otsu K. Wilson H. R. Wilson M. E. Donelson J. E. (2007). A function for a specific zinc metalloprotease of African trypanosomes. *PLoS Pathog.* 3:e150 10.1371/journal.ppat.0030150 PMC2034397 17953481

[B20] Greenhouse B. Ho B. Hubbard A. Njama-Meya D. Narum D. L. Lanar D. E. (2011). Antibodies to *Plasmodium falciparum* antigens predict a higher risk of malaria but protection from symptoms once parasitemic. *J. Infect. Dis.* 204 19–26. 10.1093/infdis/jir223 21628654 PMC3105040

[B21] Griffin J. E. Gawronski J. D. Dejesus M. A. Ioerger T. R. Akerley B. J. Sassetti C. M. (2011). High-resolution phenotypic profiling defines genes essential for mycobacterial growth and cholesterol catabolism. *PLoS Pathog.* 7:e1002251 10.1371/journal.ppat.1002251 PMC3182942 21980284

[B22] Grosset J. (2003). *Mycobacterium tuberculosis* in the extracellular compartment: an underestimated adversary. *Antimicrob. Agents Chemother.* 47 833–836. 10.1128/AAC.47.3.833-836.2003 12604509 PMC149338

[B23] Hall R. M. Ratledge C. (1982). A simple method for the production of mycobactin, the lipid-soluble siderophore, from mycobacteria. *FEMS Microbiol. Lett.* 15 133–136. 10.1111/j.1574-6968.1982.tb00053.x

[B24] Han Y. Cutler J. E. (1995). Antibody response that protects against disseminated candidiasis. *Infect. Immun.* 63 2714–2719. 7790089 10.1128/iai.63.7.2714-2719.1995 PMC173363

[B25] Hase C. C. Finkelstein R. A. (1993). Bacterial extracellular zinc-containing metalloproteases. *Microbiol. Rev.* 57 823–837. 8302217 10.1128/mr.57.4.823-837.1993 PMC372940

[B26] Jansky L. Reymanova P. Kopecky J. (2003). Dynamics of cytokine production in human peripheral blood mononuclear cells stimulated by LPS or infected by *Borrelia*. *Physiol. Res.* 52 593–598. 14535835

[B27] Johansen P. Fettelschoss A. Amstutz B. Selchow P. Waeckerle-Men Y. Keller P. (2011). Relief from Zmp1-mediated arrest of phagosome maturation is associated with facilitated presentation and enhanced immunogenicity of mycobacterial antigens. *Clin. Vaccine Immunol.* 18 907–913. 10.1128/CVI.00015-11 21471301 PMC3122614

[B28] Kelkar D. S. Kumar D. Kumar P. Balakrishnan L. Muthusamy B. Yadav A. K. (2011). Proteogenomic analysis of *Mycobacterium tuberculosis* by high resolution mass spectrometry. *Mol. Cell. Proteomics* 10 M111011627 10.1074/mcp.M111.011445 PMC3275902 21969609

[B29] Lawn S. D. Zumla A. I. (2012). Diagnosis of extrapulmonary tuberculosis using the Xpert((R)) MTB/RIF assay. *Expert Rev. Anti Infect. Ther.* 10 631–635. 10.1586/eri.12.43 22734954 PMC3605769

[B30] Malen H. Berven F. S. Fladmark K. E. Wiker H. G. (2007). Comprehensive analysis of exported proteins from *Mycobacterium tuberculosis* H37Rv. *Proteomics* 7 1702–1718. 10.1002/pmic.200600853 17443846

[B31] Malen H. De Souza G. A. Pathak S. Softeland T. Wiker H. G. (2011). Comparison of membrane proteins of *Mycobacterium tuberculosis* H37Rv and H37Ra strains. *BMC Microbiol.* 11:18 10.1186/1471-2180-11-18 PMC3033788 21261938

[B32] Malen H. Softeland T. Wiker H. G. (2008). Antigen analysis of *Mycobacterium tuberculosis* H37Rv culture filtrate proteins. *Scand. J. Immunol.* 67 245–252. 10.1111/j.1365-3083.2007.02064.x 18208443

[B33] Master S. S. Rampini S. K. Davis A. S. Keller C. Ehlers S. Springer B. (2008). *Mycobacterium tuberculosis* prevents inflammasome activation. *Cell Host Microbe* 3 224–232. 10.1016/j.chom.2008.03.003 18407066 PMC3657562

[B34] Mazandu G. K. Mulder N. J. (2012). Function prediction and analysis of *Mycobacterium tuberculosis* hypothetical proteins. *Int. J. Mol. Sci.* 13 7283–7302. 10.3390/ijms13067283 22837694 PMC3397526

[B35] Miyoshi S. Shinoda S. (2000). Microbial metalloproteases and pathogenesis. *Microbes Infect.* 2 91–98. 10.1016/S1286-4579(00)00280-X 10717546

[B36] Mori M. Moraca F. Deodato D. Ferraris D. M. Selchow P. Sander P. (2014). Discovery of the first potent and selective *Mycobacterium tuberculosis* Zmp1 inhibitor. *Bioorg. Med. Chem. Lett.* 24 2508–2511. 10.1016/j.bmcl.2014.04.004 24767848

[B37] Niki M. Suzukawa M. Akashi S. Nagai H. Ohta K. Inoue M. (2015). Evaluation of humoral immunity to *Mycobacterium tuberculosis*-specific antigens for correlation with clinical status and effective vaccine development. *J. Immunol. Res.* 2015 527395 10.1155/2015/527395 PMC4629042 26568961

[B38] Petrera A. Amstutz B. Gioia M. Hahnlein J. Baici A. Selchow P. (2012). Functional characterization of the *Mycobacterium tuberculosis* zinc metallopeptidase Zmp1 and identification of potential substrates. *Biol. Chem.* 393 631–640. 10.1515/hsz-2012-0106 22944667

[B39] Reis M. C. Rabahi M. F. Kipnis A. Junqueira-Kipnis A. P. (2009). Health care workers humoral immune response against GLcB, MPT51 and HSPX from *Mycobacterium tuberculosis*. *Braz. J. Infect. Dis.* 13 417–421. 10.1590/S1413-86702009000600006 20464332

[B40] Rowland S. S. Ruckert J. L. Burall B. N. Jr. (1997). Identification of an elastolytic protease in stationary phase culture filtrates of *M. tuberculosis*. *FEMS Microbiol. Lett.* 151 59–64. 10.1111/j.1574-6968.1997.tb10394.x 9198282

[B41] Schneider J. S. Sklar J. G. Glickman M. S. (2014). The Rip1 protease of *Mycobacterium tuberculosis* controls the SigD regulon. *J. Bacteriol.* 196 2638–2645. 10.1128/JB.01537-14 24816608 PMC4097585

[B42] Singh K. K. Dong Y. Belisle J. T. Harder J. Arora V. K. Laal S. (2005). Antigens of *Mycobacterium tuberculosis* recognized by antibodies during incipient, subclinical tuberculosis. *Clin. Diagn. Lab. Immunol.* 12 354–358. 10.1128/CDLI.12.2.354-358.2005 15699433 PMC549317

[B43] Sinha S. Kosalai K. Arora S. Namane A. Sharma P. Gaikwad A. N. (2005). Immunogenic membrane-associated proteins of *Mycobacterium tuberculosis* revealed by proteomics. *Microbiology* 151 2411–2419. 10.1099/mic.0.27799-0 16000731

[B44] Stallings C. L. Glickman M. S. (2010). Is *Mycobacterium tuberculosis* stressed out? A critical assessment of the genetic evidence. *Microbes Infect.* 12 1091–1101. 10.1016/j.micinf.2010.07.014 20691805 PMC3587153

[B45] Steingart K. R. Dendukuri N. Henry M. Schiller I. Nahid P. Hopewell P. C. (2009). Performance of purified antigens for serodiagnosis of pulmonary tuberculosis: a meta-analysis. *Clin. Vaccine Immunol.* 16 260–276. 10.1128/CVI.00355-08 19052159 PMC2643545

[B46] Stewart G. R. Wernisch L. Stabler R. Mangan J. A. Hinds J. Laing K. G. (2002). Dissection of the heat-shock response in *Mycobacterium tuberculosis* using mutants and microarrays. *Microbiology* 148 3129–3138. 10.1099/00221287-148-10-3129 12368446

[B47] Tameris M. D. Hatherill M. Landry B. S. Scriba T. J. Snowden M. A. Lockhart S. (2013). Safety and efficacy of MVA85A, a new tuberculosis vaccine, in infants previously vaccinated with BCG: a randomised, placebo-controlled phase 2b trial. *Lancet* 381 1021–1028. 10.1016/S0140-6736(13)60177-4 23391465 PMC5424647

[B48] Teitelbaum R. Glatman-Freedman A. Chen B. Robbins J. B. Unanue E. Casadevall A. (1998). A mAb recognizing a surface antigen of *Mycobacterium tuberculosis* enhances host survival. *Proc. Natl. Acad. Sci. U.S.A.* 95 15688–15693. 10.1073/pnas.95.26.15688 9861031 PMC28105

[B49] Tye G. J. Lew M. H. Choong Y. S. Lim T. S. Sarmiento M. E. Acosta A. (2015). Vaccines for TB: lessons from the past translating into future potentials. *J. Immunol. Res.* 2015 916780 10.1155/2015/916780 PMC4469767 26146643

[B50] van Crevel R. Ottenhoff T. H. van der Meer J. W. (2002). Innate immunity to *Mycobacterium tuberculosis*. *Clin. Microbiol. Rev.* 15 294–309. 10.1128/CMR.15.2.294-309.2002 11932234 PMC118070

[B51] Vecchiarelli A. Pericolini E. Gabrielli E. Pietrella D. (2012). New approaches in the development of a vaccine for mucosal candidiasis: progress and challenges. *Front. Microbiol.* 3:294 10.3389/fmicb.2012.00294 PMC3417234 22905033

[B52] Voskuil M. I. Bartek I. L. Visconti K. Schoolnik G. K. (2011). The response of *Mycobacterium tuberculosis* to reactive oxygen and nitrogen species. *Front. Microbiol.* 2:105 10.3389/fmicb.2011.00105 PMC3119406 21734908

[B53] Weiner J. III Kaufmann S. H. (2014). Recent advances towards tuberculosis control: vaccines and biomarkers. *J. Intern. Med.* 275 467–480. 10.1111/joim.12212 24635488 PMC4238842

[B54] Wen X. He L. Chi Y. Zhou S. Hoellwarth J. Zhang C. (2011). Dynamics of Th17 cells and their role in *Schistosoma japonicum* infection in C57BL/6 mice. *PLoS Negl. Trop. Dis.* 5:e1399 10.1371/journal.pntd.0001399 PMC3216943 22102924

[B55] Wu X. Yang Y. Zhang J. Li B. Liang Y. Zhang C. (2010). Comparison of antibody responses to seventeen antigens from *Mycobacterium tuberculosis*. *Clin. Chim. Acta* 411 1520–1528. 10.1016/j.cca.2010.06.014 20599865

[B56] Zhao S. Shi J. Zhang C. Zhao Y. Mao F. Yang W. (2011). Monoclonal antibodies against a *Mycobacterium tuberculosis* Ag85B-Hsp16.3 fusion protein. *Hybridoma (Larchmt)* 30 427–432. 10.1089/hyb.2011.0047 22008069

