# Supplementary figures and images for: Mycobacterium tuberculosis Zinc Metalloprotease-1 Elicits Tuberculosis-Specific Humoral Immune Response Independent of Mycobacterial Load in Pulmonary and Extra-Pulmonary Tuberculosis Patients
Source: Front Microbiol. 2016 Mar 31;7:418. doi: 10.3389/fmicb.2016.00418 (PMC4814508; doi:10.3389/fmicb.2016.00418)

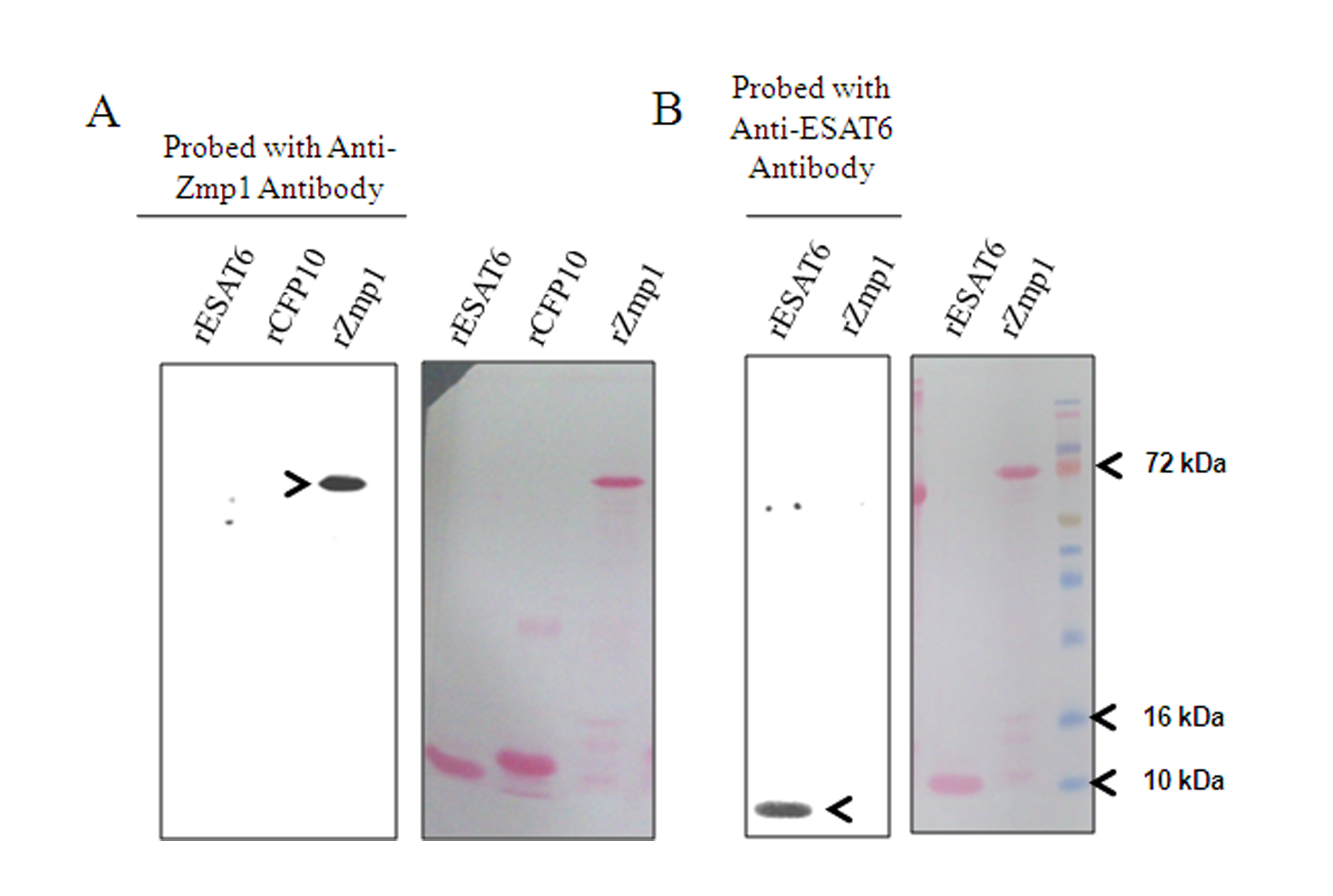

Supplement: Supplementary file 1 [file Image_1.TIF]
