# Supplementary material for: Mycobacterium tuberculosis Zinc Metalloprotease-1 Elicits Tuberculosis-Specific Humoral Immune Response Independent of Mycobacterial Load in Pulmonary and Extra-Pulmonary Tuberculosis Patients
Source: Front Microbiol. 2016 Mar 31;7:418. doi: 10.3389/fmicb.2016.00418 (PMC4814508; doi:10.3389/fmicb.2016.00418)
Supplement: Supplementary file 2 [file Table_1.DOCX]

**Supplementary Table 1:**

The table represents the results of TB Interferon Gamma Release Assay of the healthy donors used in this study. QuantiFERON®-TB Gold (QFT®) ELISA kit (Reference# 0594-0201) was used and the results were analysed using QuantiFERON-TB Gold Analysis software (Version 2.62) as per the manufacturer’s instructions. The subjects tested positive in TB IGRA test were shaded. NA represents Not Available.

|  | **Units (IU/mL)** | | | | |  |
| --- | --- | --- | --- | --- | --- | --- |
| **Subject ID** | **Nil** | **TB Ag** | **Mitogen** | **TB Ag-Nil** | **Mitogen-Nil** | **Result** |
| QC 1 | 0.18 | 0.28 | > 10 | 0.1 | > 10 | NEGATIVE |
| QC 2 | 0.17 | 0.16 | > 10 | -0.01 | > 10 | NEGATIVE |
| QC 3 | 0.11 | 0.13 | > 10 | 0.02 | > 10 | NEGATIVE |
| QC 4 | 0.11 | 0.15 | > 10 | 0.04 | > 10 | NEGATIVE |
| QC 5 | 0.14 | 0.21 | > 10 | 0.07 | > 10 | NEGATIVE |
| QC 6 | 0.13 | 0.12 | > 10 | -0.01 | > 10 | NEGATIVE |
| QC 7 | 0.22 | 0.58 | > 10 | 0.36 | > 10 | POSITIVE |
| QC 8 | 0.17 | 0.17 | > 10 | 0 | > 10 | NEGATIVE |
| QC 9 | 0.14 | 0.12 | > 10 | -0.02 | > 10 | NEGATIVE |
| QC 10 | 0.13 | > 10 | > 10 | > 10 | > 10 | POSITIVE |
| QC 11 | 0.11 | 0.17 | > 10 | 0.06 | > 10 | NEGATIVE |
| QC 12 | 0.18 | 0.2 | > 10 | 0.02 | > 10 | NEGATIVE |
| QC 13 | 0.11 | 0.13 | > 10 | 0.02 | > 10 | NEGATIVE |
| QC 14 | 0.15 | 0.14 | > 10 | -0.01 | > 10 | NEGATIVE |
| QC 15 | 0.12 | 0.15 | > 10 | 0.03 | > 10 | NEGATIVE |
| QC 16 | 0.33 | 0.29 | > 10 | -0.04 | > 10 | NEGATIVE |
| QC 17 | 0.14 | 0.13 | > 10 | -0.01 | > 10 | NEGATIVE |
| QC 18 | 0.11 | 0.12 | > 10 | 0.01 | > 10 | NEGATIVE |
| QC 19 | 0.19 | 0.13 | > 10 | -0.06 | > 10 | NEGATIVE |
| QC 20 | 0.15 | 0.13 | > 10 | -0.02 | > 10 | NEGATIVE |
| QC 21 | 0.11 | 0.11 | > 10 | 0 | > 10 | NEGATIVE |
| QC 22 | 0.11 | 0.14 | > 10 | 0.03 | > 10 | NEGATIVE |
| QC 23 | 0.13 | 0.17 | > 10 | 0.04 | > 10 | NEGATIVE |
| QC 24 | 0.19 | 0.13 | > 10 | -0.06 | > 10 | NEGATIVE |
| QC 25 | 0.12 | 0.15 | > 10 | 0.03 | > 10 | NEGATIVE |
| QC 26 | 0.16 | 0.13 | > 10 | -0.03 | > 10 | NEGATIVE |
| QC 27 | 0.11 | 0.12 | > 10 | 0.01 | > 10 | NEGATIVE |
| QC 28 | 0.11 | > 10 | > 10 | > 10 | > 10 | POSITIVE |
| QC 29 | 0.17 | 2.67 | > 10 | 2.5 | > 10 | POSITIVE |
| QC 30 | 0.12 | 0.1 | > 10 | -0.02 | > 10 | NEGATIVE |
| QC 31 | 0.19 | 5.78 | > 10 | 5.59 | > 10 | POSITIVE |
| QC 32 | 0.13 | 1.1 | > 10 | 0.97 | > 10 | POSITIVE |
| QC 33 | 0.23 | 0.2 | > 10 | -0.03 | > 10 | NEGATIVE |
| QC 34 | 0.17 | 0.22 | > 10 | 0.05 | > 10 | NEGATIVE |
| QC 35 | 0.13 | 0.28 | > 10 | 0.15 | > 10 | NEGATIVE |
| QC 36 | 0.17 | 0.13 | > 10 | -0.04 | > 10 | NEGATIVE |
| QC 37 | NA | NA | NA | NA | NA | NA |
| QC 38 | 0.13 | 0.23 | > 10 | 0.1 | > 10 | NEGATIVE |
| QC 39 | 0.12 | 0.13 | > 10 | 0.01 | > 10 | NEGATIVE |
| QC 40 | 0.12 | 0.12 | > 10 | 0 | > 10 | NEGATIVE |
| QC 41 | 0.13 | 0.13 | > 10 | 0 | > 10 | NEGATIVE |
| QC 42 | 0.13 | 0.17 | > 10 | 0.04 | > 10 | NEGATIVE |
| QC 43 | 0.15 | 0.15 | > 10 | 0 | > 10 | NEGATIVE |
| QC 44 | 0.12 | 0.14 | > 10 | 0.02 | > 10 | NEGATIVE |
| QC 45 | 0.16 | 0.15 | > 10 | -0.01 | > 10 | NEGATIVE |
| QC 46 | 0.12 | 0.11 | > 10 | -0.01 | > 10 | NEGATIVE |
| QC 47 | 0.26 | 0.33 | > 10 | 0.07 | > 10 | NEGATIVE |
| QC 48 | 0.13 | 0.12 | > 10 | -0.01 | > 10 | NEGATIVE |
| QC 49 | 0.14 | 0.16 | > 10 | 0.02 | > 10 | NEGATIVE |
| QC 50 | 0.2 | 0.17 | > 10 | -0.03 | > 10 | NEGATIVE |
| QC 51 | 0.13 | 0.17 | > 10 | 0.04 | > 10 | NEGATIVE |
| QC 52 | 0.14 | 0.13 | > 10 | -0.01 | > 10 | NEGATIVE |
| QC 53 | 0.11 | 0.2 | > 10 | 0.09 | > 10 | NEGATIVE |
| QC 54 | 0.18 | 0.21 | > 10 | 0.03 | > 10 | NEGATIVE |
| QC 55 | 0.44 | 0.29 | 7.99 | -0.15 | 7.55 | NEGATIVE |
| QC 56 | 0.14 | 0.59 | > 10 | 0.45 | > 10 | POSITIVE |
| QC 57 | 0.13 | 0.21 | > 10 | 0.08 | > 10 | NEGATIVE |
| QC 58 | 0.16 | 0.21 | > 10 | 0.05 | > 10 | NEGATIVE |
| QC 59 | 0.13 | > 10 | > 10 | > 10 | > 10 | POSITIVE |
| QC 60 | 0.12 | 0.14 | > 10 | 0.02 | > 10 | NEGATIVE |
| QC 61 | 0.15 | 0.14 | > 10 | -0.01 | > 10 | NEGATIVE |
| QC 62 | 0.14 | 2.68 | > 10 | 2.54 | > 10 | POSITIVE |
